# Serum Metabolic Profile Alteration Reveals Response to Platinum-Based Combination Chemotherapy for Lung Cancer: Sensitive Patients Distinguished from Insensitive ones

**DOI:** 10.1038/s41598-017-16085-y

**Published:** 2017-12-13

**Authors:** Shan Xu, Yanping Zhou, Hui Geng, Dandan Song, Jing Tang, Xianmin Zhu, Di Yu, Sheng Hu, Yanfang Cui

**Affiliations:** 1 Key Laboratory of Pesticide and Chemical Biology, Ministry of Education, Central China Normal University, Wuhan, 430079 P. R. China; 2 Department of Medical Oncology, Hubei Province Tumor Hospital, Wuhan, 430079 P.R. China; 3 Department of Life Sciences, Central China Normal University, Wuhan, 430079 P. R. China; 4 Tongji Medical College, Huazhong University of Science and Technology, Wuhan, 430030 P.R. China; 5 CAS Key Laboratory of Magnetic Resonance in Biological Systems, State Key Laboratory of Magnetic Resonance and Atomic and Molecular Physics, National Centre for Magnetic Resonance in Wuhan, Wuhan Institute of Physics and Mathematics, University of Chinese Academy of Sciences, Wuhan, 430071 China; 6 Department of Biochemistry and Molecular Biology, Biomedicine Discovery Institute, Monash University, Clayton VIC 3800, Australia

## Abstract

Most lung cancers are diagnosed at fairly advanced stages due to limited clinical symptoms. Platinum-based chemotherapy, either as single regimen or in combination with radiation, is one of the major recommendations for the patients. Earlier evaluation of the effectiveness of the chemotherapies is critical for developing better treatment plan given the toxicity of the chemotherapeutic reagents. Drug efficacy could be reflected in the systemic metabolism characteristics though knowledge about which remains scarce. In this study, serum metabolism influence of three types of commonly used platinum-based combination chemotherapy regimens, namely cisplatin with gemcitabine, vinorelbine or docetaxel, were studied using pattern recognition coupled with nuclear magnetic resonance techniques. The treated patients were divided into sensitive or insensitive subgroups according to their response to the treatments. We found that insensitive subjects can be identified from the sensitive ones with up-regulation of glucose and taurine but reduced alanine and lactate concentrations in serum. The combination chemotherapy of lung cancer is accompanied by disturbances of multiple metabolic pathways such as energy metabolism, phosphatidylcholine biosynthesis, so that the treated patients were marginally discriminated from the untreated. Serum metabolic profile of patients shows potential as an indicator of their response to platinum-based combination chemotherapy.

## Introduction

Lung cancer is the leading cause of cancer-related deaths worldwide, with an approximate annual incidence rate of 1.5 million and a 5-year survival rate of 15%^1,2^. The majority of lung cancer cases are already locally advanced or at the metastatic stage (IIIB or IV) at initial diagnosis, thus unsuitable for surgery^3,4^. Platinum-based chemotherapy has consequently been the main therapeutic option for both advanced non-small cell lung cancer (NSCLC) and small cell lung cancer (SCLC), even though a significant proportion of patients are insensitive^5^ to such treatments. Typicaly, it often takes 6–9 weeks (2–3 cycles) platinum-based treatments to determine the response to the treatments according to Response Evaluation Criteria in Solid Tumors (RECISTv1.1), meaning a lengthy period of exposure to the therapy toxicities that often reflect in side effects such as bone marrow and renal impairment, and neurotoxicity^5^. Therefore, to identify timely and non-invasively the large population insensitive to chemotherapy is important for not only developing more suitable treatment protocols, but also reducing unnecessary exposure to the toxic drugs.

In addition to the well acknowledged factors that influence therapy sensitivity such as age, diet and liver and kidney functionality, genetic polymorphism (more commonly single nucleotide polymorphisms, SNPs) has been demonstrated to be related to the outcome of cancer treatments^6^, such that a proportion of lung cancer patients can now benefit from targeted agents such as FDA approved EGFR and ALK inhibitors^7,8^. However, gene abnormalities are uncommon and virtually all patients develop resistance to the corresponding target therapy^9^. Therefore, the majority of patients still need to go through conventional chemotherapies or chemotherapy-based combination regimens such as combination of anti-angiogenesis targeted inhibitors with chemotherapies, as a strategy to overcome drug resistancein a clinical setting, so it remains an urgency to timely evaluate the efficacy of chemotherapeutic agents.

Expression or activities of a large group of drug metabolising enzymes, drug transporters and drug target enzymes that modulate intracellular drug accumulation are relevant to platinum resistance^10^. The expression of excision repair cross-complementation group 1 (ERCC1) is another identified factor that regulates the DNA adduct level and proven to be correlated with cisplatin resistance in NSCLC^11^ although there are a few inconsistent results^12,13^. These updated understanding of the molecular determinants of chemo-drug resistance are promising but no biomarkers have been developed to predict the sensitivity of treatment. Persistent investigation beyond genetic aberrance is warranted to understand the mechanism of drug resistance as well as the consequence of lung cancer chemotherapy.

DNA replication and microtubule function influenced by most active chemotherapy agents are essential for most biological processes, including systemic metabolism. Therefore, it is conceivable that administration of therapeutic agents could likely cause alteration of metabolism pathways and thus of metabolites as the end products. A recent signalling-based study of dynamic response to platinum drugs and taxanes have proved that HIF1a, CHEK1 and CHEK2, as well as some other genes for DNA repair, cell cycle and apoptosis were up-regulated in chemotherapy drug treated xenografts^14^, suggesting a driving effect on these pathways immediately after treatment administration^15^. To further the signalling pathway study to the level of metabolism, we ask if the affected signalling pathways converge to adapt tumor cell metabolism to cope with the impact of the drugs and thus resulting in a certain pattern of metabolism alteration, and if we can find out any metabolic hallmarks in association with the conventional chemotherapy treatments.

The rapidly advancing metabolomics techniques displaying simultaneous observation of multiple markers^16,17^, in combination with multivariate analysis providing enhanced interpretation of high information content for pathological conditions with great complexity, have successfully identified biomarkers for several types of human cancers^18,19^ and increasing number of fingerprints for treatment response and toxicity^19–21^. Metabolism consequence of lung cancer targeted therapies has been characterized^22^; a study on human A549 cells treated with cisplatin suggested possibility of metabolite signatures as response to chemotherapy^23^; a pilot study on 25 patients before, during and after chemotherapy ± radiation treatments indicated that serum metabolites were strongly correlative with not only cancer stage and cancer type, but also the prognosis^24^. These promising results somewhat justified the speculation that chemotherapy sensitivity might be elicited in drug-caused metabolism alteration even with the multifactorial nature of chemotherapy resistance and the lack of understanding on the impact of each factors.

In this study, we focus on more general clinical settings where combination chemotherapy regimens are applied. Different drugs are often concurrently used in belief that it would be more difficult for cancer cells to develop resistance. The serum metabolite profile of lung cancer patients who went through three kinds of platinium-based combination chemotherapy regimens, that is cisplatin with three microtubule regulating reagents namely gemcitabine, vinorelbine, or docetaxel, were studied using pattern recognition coupled with nuclear magnetic resonance (NMR) techniques. When analysing with validated OPLS-DA models, patients that are sensitive or insensitive to the treatments can be discriminated with different metabolite profiles. Lung cancer serum after treatment is accompanied by disturbances of multiple metabolic pathways, including alterations in energy metabolism and phosphatidylcholine biosynthesis.

## Results

### NMR Spectra Revealed Metabolite Differences between Healthy, Untreated And Treated Subjects

^1^H NMR spectra of serum samples from healthy and cancerous people displayed signals of twenty-seven metabolites including amino acids, lactate, choline-containing, lipid, glucose, pyruvate, trimethylamine and nucleic acids, and all were unambiguously assigned (Table S1) according to previous studies and a series of 2D NMR spectra. Obvious metabolite differences between the three groups can be visualized from ^1^H NMR spectra (Figure S1).

### Serum Metabolomic Characteristics of the Subjects

**Figure 1:**
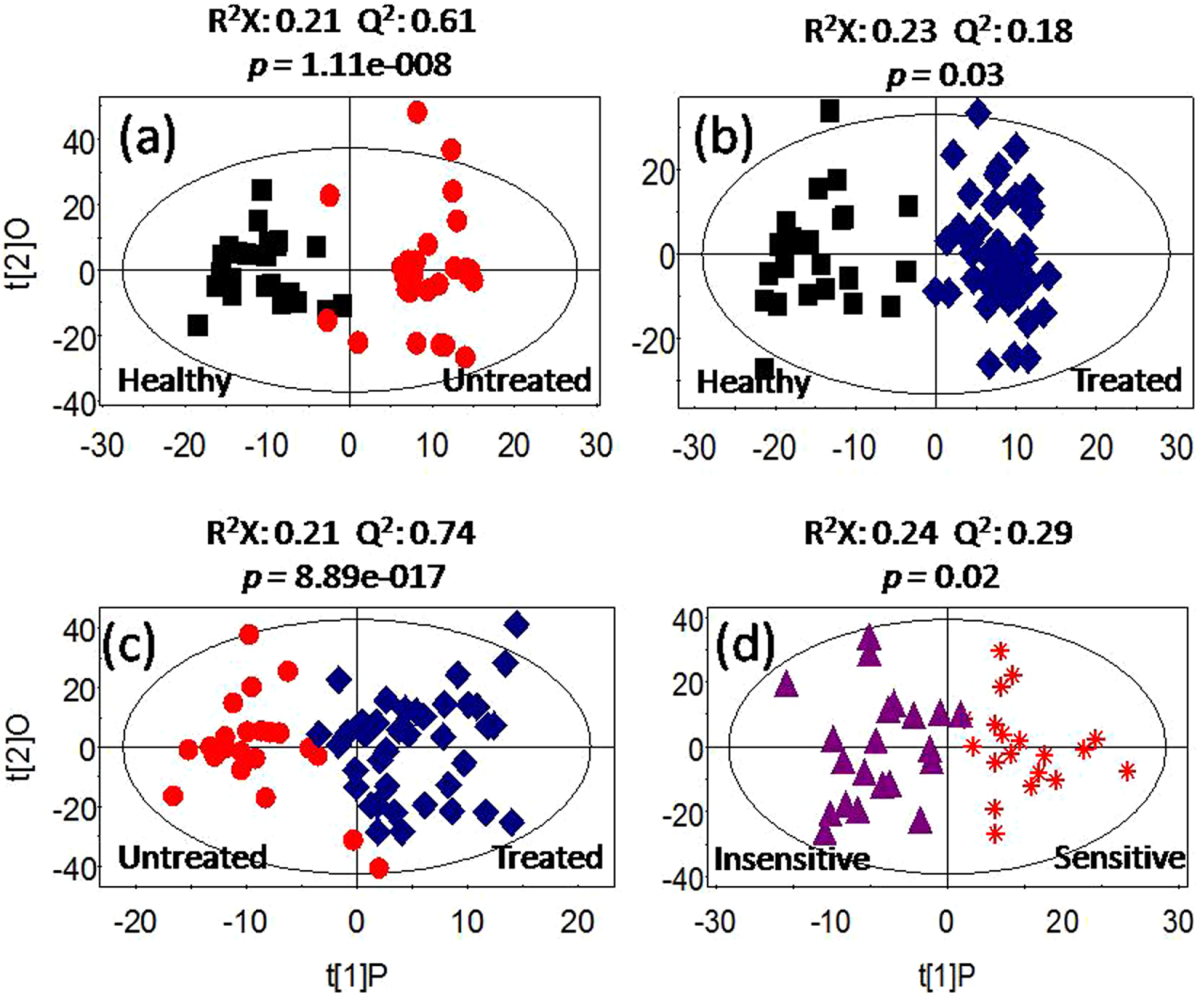
OPLS-DA scores showing the discrimination between groups of healthy control (■), Untreated (), Treated (), Insensitive () and Sensitive ().

Unsupervised principal component analysis (PCA) was conducted on the ^1^H NMR data to detect possible direct classification and/or outliers. Supervised orthogonal project to latent structure-discriminant analysis (OPLS-DA) was further employed to improve classification and decipher the serum metabolite profiles. The PCA results showed no outliers and clear separation was indicated between the healthy group and the treated group (healthy *vs* treated, Figure S2a) as well as healthy *vs* untreated (Figure S2b). As for the comparison of treated *vs* untreated, or sensitive *vs* insensitive, unsupervised discrimination showed no success (Figure S2c,d). However, OPLS-DA classification models were established for every comparison inter-groups (Fig. 1), indicating discriminative metabolite profiles between the inter-groups. To evaluate the validity of the supervised models, t-test was executed with CV-ANOVA approach and the results confirmed the statistical significance (p < 0.05) for these models (Fig. 1). Quality of supervised models for groups that were compared with each other and the identified differences in metabolites are listed as follows.

#### Healthy vs Untreated

**Figure 2:**
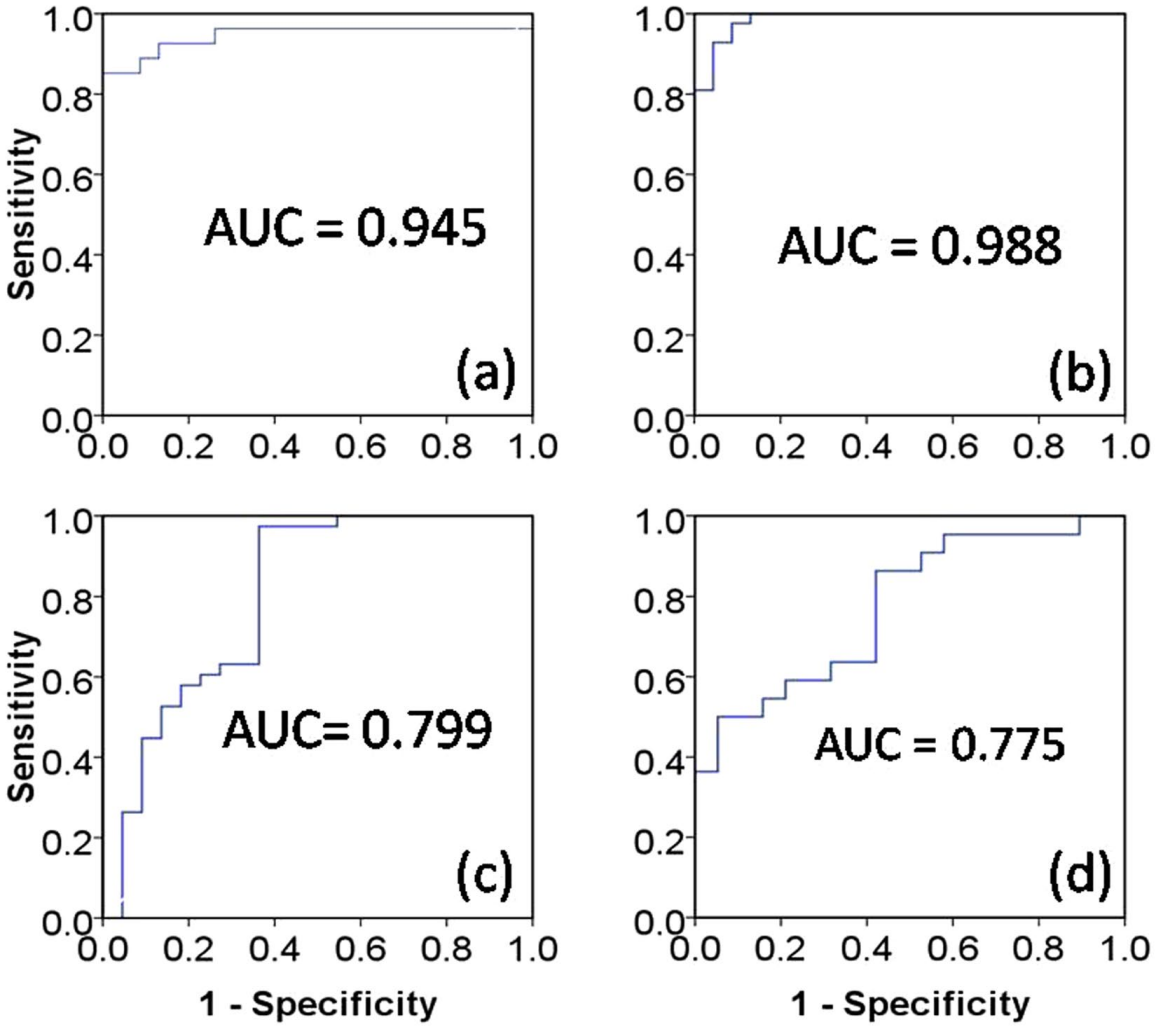
ROC curves determined using the cross-validated predicted Y-values of the ^1^H NMR OPLS-DA models, (**a**) healthy *vs* untreated, (**b**) healthy *vs* treated, (**c**) untreated *vs* treated, (**d**) sensitive *vs* insensitive.

**Figure 3:**
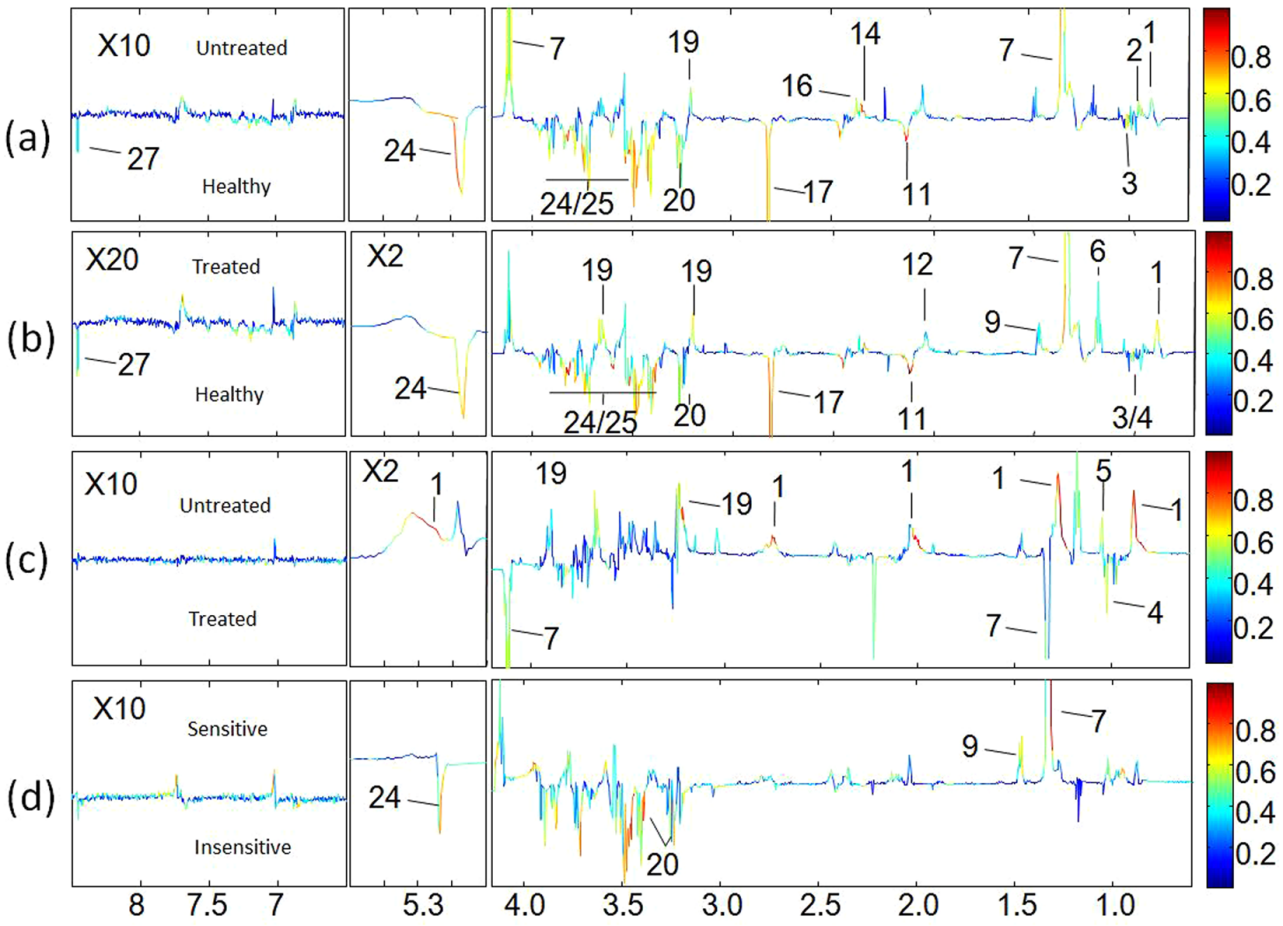
Corresponding loadings plots derived from the ^1^H NMR data for serum between groups of healthy control, treated and untreated, sensitive and insensitive. Key parameters of metabolites are given in Table S1.

OPLS-DA model quality (R^2^X = 0.21, Q2 = 0.61) (Fig. 1a) between the two groups revealed clear differentiation between the healthy and the untreated groups. Receiver operating characteristic (ROC) analysis further confirmed the result (Fig. 2a) with the area under the curve (AUC) being 0.94, indicating good diagnostic power of the model. Compared with the healthy group, untreated cancer sera contained higher levels of lipid, isoleucine, lactate, glutamine, pyruvate and choline, and lower levels of leucnine, methionine, trimethylamine, taurine, glucose, and formate as the loadings plots showed (Fig. 3a).

#### Healthy vs Treated

OPLS-DA model quality (R^2^X = 0.23, Q2 = 0.18) (Fig. 1b) revealed clear differentiation between the serum metabolites of the control and the treated groups. The AUC is 0.98 with ROC analysis (Fig. 2b), which confirmed the sharp distinction between the two groups. Corresponding loadings plots showed higher levels of lipid, lactate, 3-hydroxybutyrate, alanine, glycoprotein (acetyls) and choline, and lower levels of leucine, valine, methionine, trimethylamine, taurine, glucose and formate in the treated serum (Fig. 3b) compared to the healthy people.

#### Treated vs Untreated

To evaluate the treatment effect of chemotherapy regimens, the metabolic profiles between the untreated and the treated groups were compared. The OPLS-DA comparison model was verified with T-test (p < 0.05) but with modest model quality (R^2^X = 0.21, Q2 = 0.74) (Fig. 1c), AUC is 0.80 with ROC analysis indicated a modest distinction between the treated and the untreated cancer serum (Fig. 2c). Compared to the untreated cancer serum, higher levels of lipid, isobutyrate and choline, and lower levels of valine and lactate were found in treated serum (Fig. 3c).

#### Sensitive vs Insensitive

In the treated group, 22 patients are defined as sensitive to the treatments and 20 patients not sensitive (See materials and methods section). OPLS-DA model quality (R^2^X = 0.24, Q2 = 0.29) and T-test (p < 0.05) revealed that the sensitive patients could be well distinguished from the insensitive treatment group (Fig. 1d). The AUC is 0.77, indicating that the model predictive ability is quite strong (Fig. 2d). Corresponding loadings plots of the serum metabolites consistently revealed significant differences between sensitive and insensitive treatment groups. Compared to the insensitive group, there are higher levels of lactate and alanine, but lower levels of taurine and glucose in the sensitive groups (Fig. 3d).

It can be seen that treatment of lung cancer caused changes in several metabolic pathways compared with the untreated or healthy, including energy metabolism (glycolysis and lipid), phosphatidylcholine biosynthesis as well as amino acid metabolism; alteration in similar spectrum of pathways was also observed between untreated *vs* healthy. Whereas, the difference between sensitive and insensitive groups is manifested mainly in energy metabolism.

### Relative Concentrations of Metabolites among Cancer, Treated and Healthy Serum

**Figure 4:**
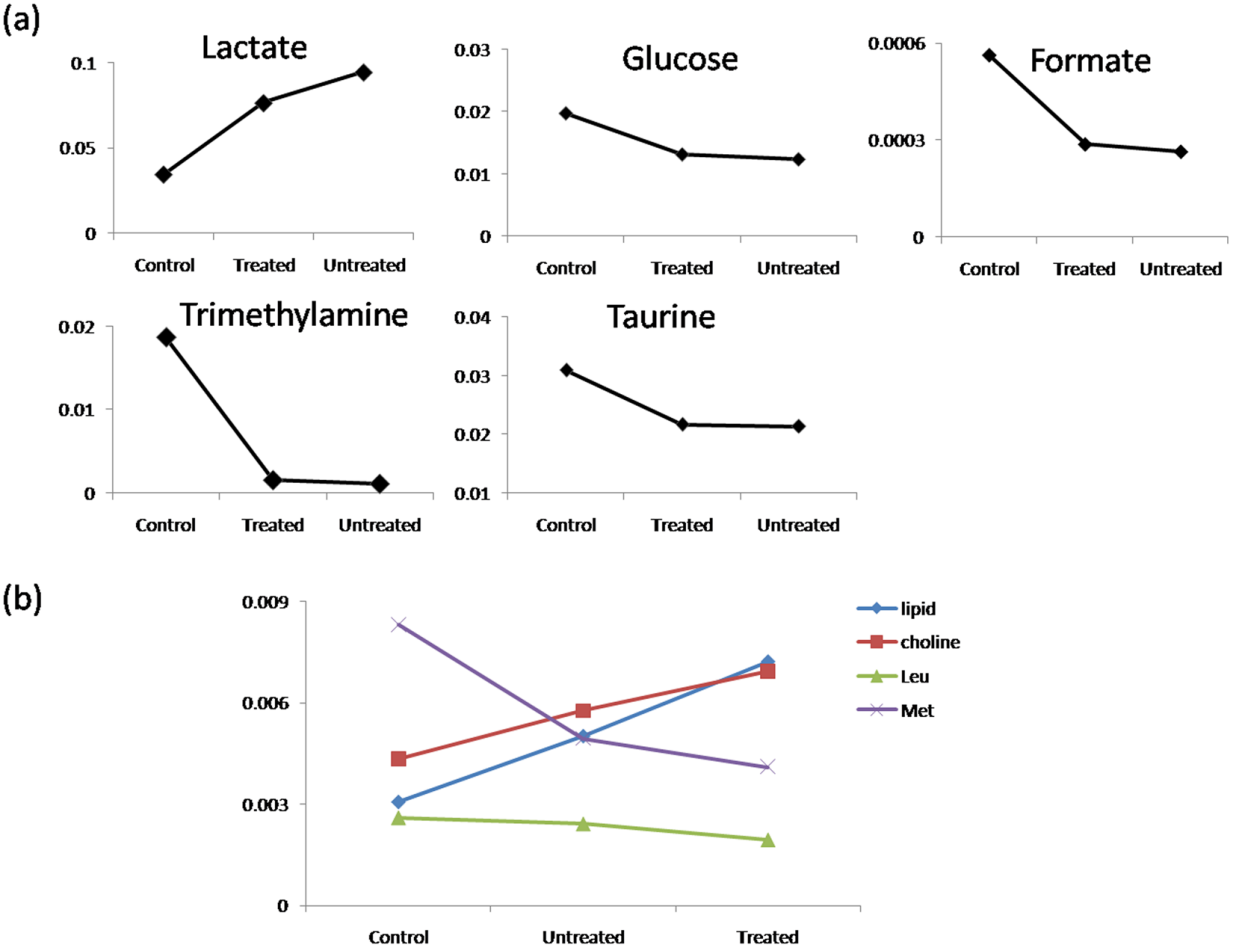
Relative metabolite concentrations in serum among the healthy, treated and untreated groups.

The serum metabolites of the untreated, treated and healthy were compared to explore the relative metabolite concentrations among the three populations. The relative quantification of the metabolites was obtained by comparing the integrals of the corresponding characteristic metabolite peaks in NMR spectra, and only the characteristic NMR peaks of the metabolites showing the least overlap were integrated. The results showed that the treated patients present much lower level of lactate than the cancer group, but still twice that of the healthy group, thus the untreated patients displayed the highest level of lactate amongst the three groups. On the other hand, the treatments slightly increased the levels of TMA, taurine, glucose and formate compared to the untreated, but dramatically lower than that of the normal control (Fig. 4a), suggesting that the chemotherapy treatment altered the metabolism of cancer and made it closer to that of the healthy.

Consisting with the notion that chemotherapy drugs could exert dramatic effects on the cell destruction, we observed that the highest levels of lipid and choline while the lowest levels of leucine and methionine in the treated group (Fig. 4b). This relative concentration levels indicated that tumor burden increased the metabolism activity related to these metabolites, but chemotherapy influence on the corresponding metabolism is more dramatic.

## Discussion

Most lung cancer patients are confronted with late diagnosis and low cure rate. The majority of the patients who have no abnormal genetic biomarkers would probably go through 4–6 cycles of standard either adjuvant or neo-adjuvant chemotherapy treatments with each cycle lasting 2–3 weeks. Currently there is no other approach than morphological measurements to know ahead of time who is going to benefit. Recent active investigation and advances in the target therapy, immune checkpoints inhibitory pathway blocking and other immunotherapies such as chimeric antigen receptor T cell strategy can only benefit a small fraction of patients partly due to the heterogeneous and complex solid tumor microenvironment. Combination of these new therapeutic methods with conventional chemotherapy to improve the therapeutic efficacy is under exploration and appears to be promising^25,26^. As a result, platinum-based therapy in combination with the second or third generation agents is currently the mainstay in lung cancer treatment, nowadays and probably in the few years to come. Up to date, little is known about the overall metabolite profiles of lung cancer patients under platinum-based combination chemotherapy although this biological information is of potential significance for understanding metabolism influence of chemotherapy to human body, and from which metabonomics approaches may be developed to assist the evaluation of the conventional treatment for lung cancer.

In this study we outlined the serum metabolites pattern of lung cancer patients who went through three platinum-based combination chemotherapy regimens based on a hypothesis that the patients accepted standard chemotherapy treatments might display distinct serum metabolite profile compared with the untreated patients, especially the sensitive might be able to be identified from those insensitive. The results clearly demonstrated that sensitive patients can be identified from insensitive patients, and the treated lung cancer population exhibited distinctive serum metabolic characteristics with respect to the untreated lung cancer people. Meanwhile, both untreated and treated lung cancer cohorts can be distinguished from the healthy controls with high specificity and sensitivity. Since serum metabolites profile reflect comprehensive metabolic characteristic information and the body metabolic status in response to multifaceted factors, these information can potentially be used for treatment prognosis of lung cancer, which could provide potential guides for clinical management of lung cancer.

### Metabolic Characteristics of Untreated Lung Cancer: Healthy vs Untreated

As the basis of drug response study, we first compared the metabolic differences of untreated and healthy serum (Fig. 1a). The results are mostly consistent with related earlier studies of ours^27^ and others^28,29^. Significantly enhanced energy metabolism activity was clearly observed from higher level of lactate and lower level of glucose in the untreated serum comparing with the healthy, coinciding with the feature of Warburg Effect that includes promoted aerobic glycolysis and high level of glucose uptake with lactate production during tumor cell proliferation^30^, an observation that was also documented for other cancers^31–33^. Increased lipogenesis (high level of lipid)^34^, increased ketone body metabolism and fatty acid oxidation (as leucine level reduced), as well as higher concentrations of pyruvate (complying with high glucolysis^31^), Isoleucine (due to reduced activity of succinyl-CoA resulted from restrained TCA cycle and enhanced glycolysis^32^), and glutamine (as energy source^35^), could also provide energy and components necessary for lung tumor growth.

Higher level of choline in untreated serum indicated increased cell membrane synthesis for tumor cell proliferation^32^, together with lower level of trimethylamine further suggested that the activity of choline oxidation into trimethylamine is restrained. Choline and methionine take part in methyl donor cycle that provides energy and necessary materials for tumor proliferation^36^. Altered level of taurine indicates that tumor proliferation induced disorder of the osmotic adjustment. Decreased level of formate in untreated lung cancer patients was also detected previously^28^.

### Metabolism Regulation Effects of Chemotherapy for Lung Cancer

All the treated patients, no matter it is sensitive or insensitive according to standard assessment, exhibited altered metabolic profiles as compared with the untreated patients. Multiple metabolite parameters contribute to the distinction between the two groups (Fig. 3c), indicating the chemotherapeutic agents caused profound metabolism shift. To examine if the metabolism characters of the treated patients were readjusted to close to that of the healthy people, the treated was also compared with the healthy, the results showed obvious difference between the two groups (Fig. 3b). Further, a few commonly identified molecules were compared among the groups as discussed in the following context to elicit underlying trends if any.

#### Treated vs Untreated

**Figure 5:**
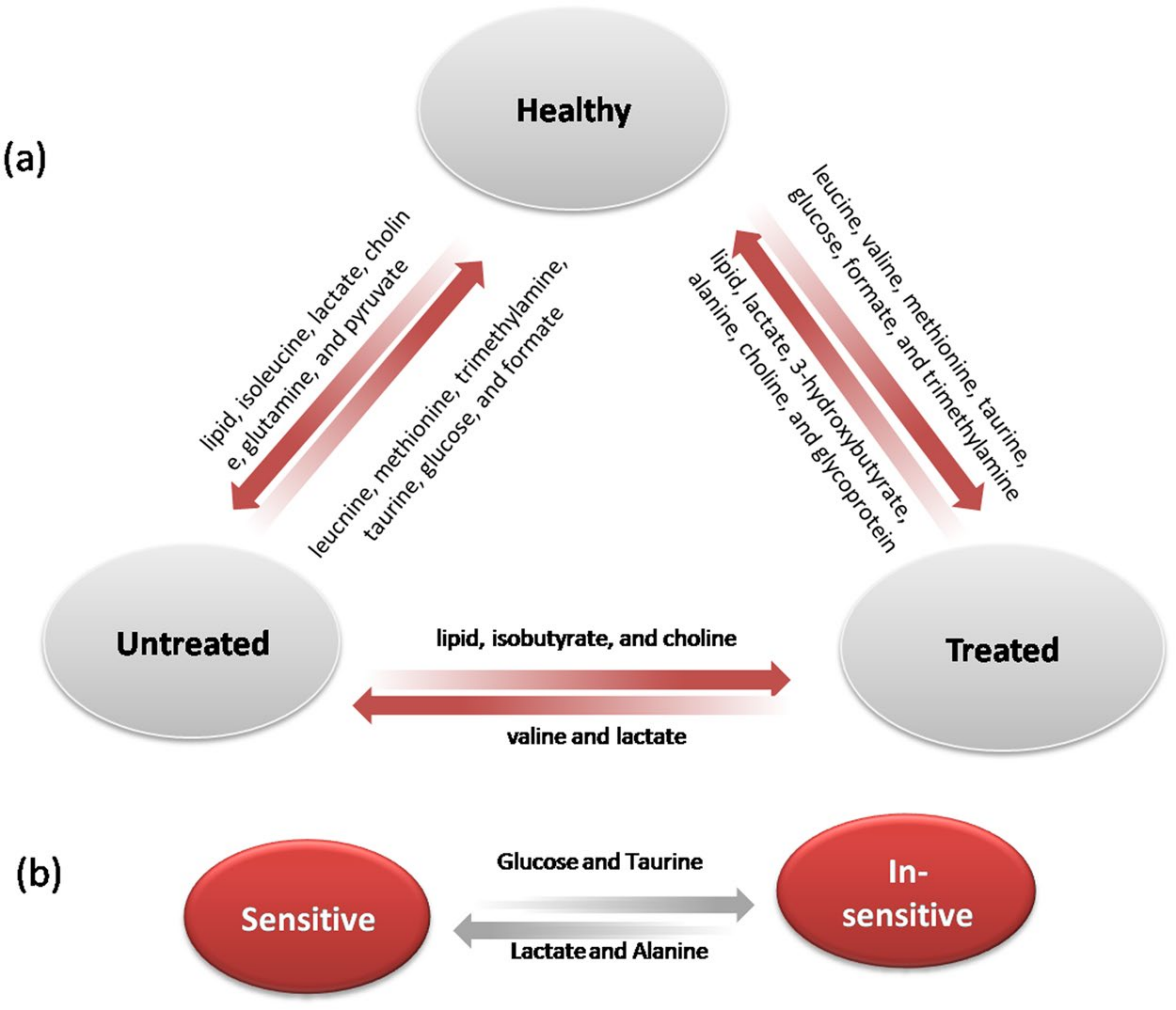
Schematic relative concentrations showing the metabolites profile that is discriminating among the treated, untreated, and healthy groups. Arrows pointing toward the groups with elevated specified metabolites.

The metabolism of lung cancer exhibited obvious change after treatment with chemotherapy drugs. Under the effect of chemotherapy, lower levels of lactate and valine illustrates that the glycolysis was getting relieved and showed the effectiveness of therapeutic agents (Fig. 5a), being consistent with the observation that target therapy agents reverse the Warburg effect. The higher levels of lipid, isobutyrate and choline in treated sera suggested that lipid and choline metabolism were affected by chemotherapy. We suppose that chemotherapy break the cell membrane and then induced lipid to be increased. A study of cisplatin-treated A549 cells also observed increase in lipids except nucleotides sugars and sorbitol, but decrease in niacinamide and amino acids^23^.

#### Treated vs Healthy

There were higher levels of lipid, lactate and choline, as well as lower levels of leucine, methionine, trimethylamine, taurine, glucose and formate intreated group compared with the healthy, which resembles the metabolites difference between the groups of treated and healthy. In addition, other increased metabolites including 3-hydroxybutyrate, alanine, glycoproteins (acetyls) and valine were also observable in treated group (Fig. 5a). Glycoproteins are made up of oligosaccharides and polypeptide chain, widely exist in cell membrane and blood plasma, functioning as important physiological active substances. The sugar chains of glycoprotein play important role in maintaining protein stability and resistance to protease hydrolysis^37^. Therefore, it is likely that chemo-drugs cause cell membrane rupture and decomposition, thereby enabling lipid and glycoproteins being released into the blood and resulting in elevated level of lipid and glycoproteins. 3-hydroxybutyrate as a ketogenesis metabolite associates with lipid metabolism, so higher level of 3-hydroxybutyrate in treated serum suggested that the chemo-drugs facilitated lipolysis. Elevated alanine (reversibly being converted into pyruvate involved in both glycolysis and TCA cycle^31,38^) and valine (can be converted to succinate) suggested that chemo-drugs are changing active glycolysis in untreated into active TCA reactions in treated for exuberant energy metabolism, but the metabolism characters after chemotherapy is closer to the untreated metabolism than the healthy.

#### Relative Metabolites Levels among Untreated, Treated and Healthy

Concentrations among the three groups of healthy, treated and untreated revealed that relative extents of the metabolism influence from tumor growth and chemotherapy drugs. The declined level of lactate from diagnosis to healthy serum suggested that glycolysis was restrained after chemotherapy but still higher than the healthy serum. Meanwhile, the levels of glucose, taurine, trimethylamine and formate elevated after chemotherapy but still below the healthy level (Fig. 4). This metabolism information reflected that the chemotherapy resulted in certain therapeutic effect on lung cancer, such as reversed the Warburg effect to some extent, but still in pathologic stage compared with the healthy. Converse to elevated glycolytic rates in tumors, decrease in conversion of pyruvate to lactate in response to treatment with phosphoinositide 3-kinase (PI3K) or receptor tyrosine kinase (RTK) inhibitors was shown in different tumor types^39,40^.

Compared to the healthy serum, levels of methionine and leucine reduced in the untreated lung cancer serum, and became even lower after treatment, implying that methionine and leucine were highly consumed as nutrients for the recovery after treated with chemotherapy. Alanine, a kind of similar building materials of protein, was observed being decreased in chemotherapy insensitive patients contrast to the sensitive ones, a phenomenon that can be elucidated by the fact that insensitive patients might have higher protein synthesis demands for platinum efflux and DNA repair, the common causes of chemo-drug resistance.

In contrast, our observation of the elevated choline and lipid level in treated serum compared to either untreated or healthy group suggested that the amount of lipid and phosphatidylcholines (PtC) released from cell membrane rupture is overwhelming although the cell membrane reconstruction demands degradation of choline into PC, given the fact that lipid and choline are the main components of cell membrane. GPC is derived from PtC, followed by conversion into choline^36^. On the other hand, cancer cell proliferation demands building of cell membrane, which needs building blocks of PC and PtC, both can be converted from choline by choline kinases^41,42^. Inhibition of cell proliferation following treatment with targeted therapies, including inhibitors of Ras, PI3K, mitogen activated protein kinases (MAPK) and hypoxia inducible factor (HIF) led in most cases to a drop in PC and choline. However, treatment with the heat shock protein (HSP) 90 inhibitor was reported to cause an increase in PC in several cancer models^43^.

We recently observed that tumor-growth causes elevation of TMA in lung and liver tissues in xenograft lung cancer mice model^44^. Here we found that lung cancer patients with and without chemotherapy treatments both display lowered serum level of TMA compared with healthy, while the cancerous group and the treated group display no prominent difference in TMA level. Now it is accepted that in mammals TMA is largely generated from choline and carnitine by gut microbiota in gastrointestinal track and then absorbed into liver via enter hepatic circulation, or into lung via air-blood exchange. Therefore, our observations from human and mice model suggest that lung cancer likely influences gut microbiota functions causing TMA level enhancement in liver and lung but decrease in serum, and chemotherapy treatments exert little effect on the influence. Further research on this speculation is needed.

### Metabolites Difference between Sensitive and Insensitive Patients to Treatment

**Figure 6:**
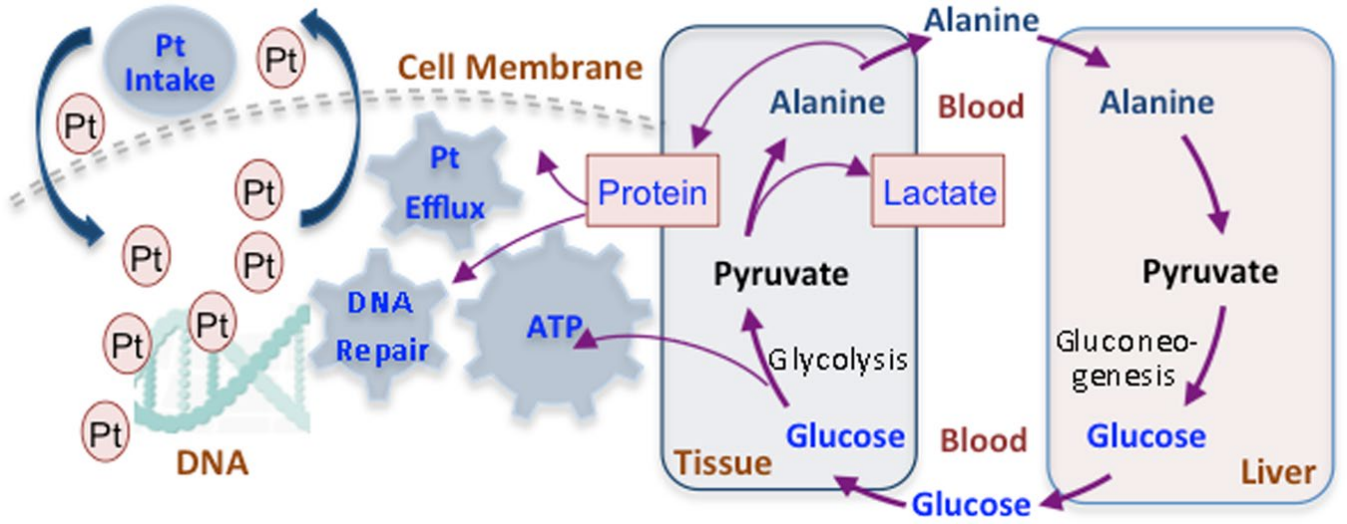
Proposed metabolism basis underlying the chemo-drug resistance. The correlated events of glycolysis, gluconeogenesis, and glucose-alanine cycle provide protein synthesis materials and energy for cellular platinum efflux and DNA repair; both actions contribute to platinum drug resistance. One mole of glucose breaks down into two mole of pyruvate, during which the net energy obtaining is two mole of ATP. These ATP can be utilized in the activity of DNA repair from the damage of platinum-DNA adducts, as well as the platinum efflux from the cell.

Chemotherapy drug resistance prevails in in-sensitive patients, the resistance is considered being caused majorly by extensive DNA-platinum adducts repair as well as cellular platinum efflux by a group of transporter proteins. Both functions probably need extra energy and expression of repairing proteins or efflux transporter proteins. The damage of DNA by bound platinum is majorly repaired by the so-called ERCC1 and its expression correlates with cisplatin resistance^11^. Glutathione (GSH) can repair adducts and perform platinum-detoxification as well^45^. Regarding platinum efflux transporter proteins, expression of ATP7B and ABCG2 was mostly responsible and proved to be correlated to the platinum-drug resistance^10^. A group of proteins such as human equilibrative nucleoside transporter 1 (hENT-1), Ribonucleotidereductase M1 and M2 (RRM1 and RRM2), and Mitogen-activated protein kinases (MAPKs) have been suggested being associated to the resistance of gemcitabine, vinerolbine or taxanes^10^. Theoretically, expression of the proteins would need amount of building blocks, i.e. amino acids. The energy needs may be largely met from glycolysis, a process that elevates lactate level and reduces glucose level, whereas, the building blocks of proteins should be generated from nutrient degradation or gluconeogenesis, both processes would provide more glucose. Insensitive patients present a phenotype of high glucose but low lactate and alanine levels compared with the sensitive ones, indicating that the chemo-resistance demanded energy and protein expression get the glucose-alanine cycle, glycolysis, and gluconeogenesis processes coupled (Fig. 5). Plausible mechanism is that the formed alanine is drained and biased into protein building channel, leading to less lactate formation and reduction of alanine level as well. Given the observation of the treated including both sensitive and insensitive displayed similar restriction in the glycolysis comparing to the untreated as stated before, the possibility that more restrained glycolysis in the in-sensitive resulting in more glucose containment but low pyruvate and thus low alanine and lactate could be ruled out. Coupling of high glucose and low alanine and lactate levels in insensitive, indicating the glucose supply from nutrients breakdown or gluconeogenesis is larger than glucose consumption by the energy needs from the efflux and excision (Fig. 6).

Insensitive patients showed elevated taurine level than the sensitive ones. As a major constituent of bile, taurine can be found in the lower intestine and is a derivative of cysteine. The sulfhydryl group of cysteine is first oxidized to cysteine sulfinic acid, which in turn is decarboxylated to form hypotaurine, reactions occur in the pancreas via the cysteine sulfinic acid pathway. In this pathway, taurine can then conjugate with chenodeoxy-cholic acid and cholic acid to form bile salts. This conversion occurs in the liver before the produced bile salts being released into bile again. Insensitive patients have more taurine in the blood, indicating conversion from bile acid to bile salts was restrained or the formation of bile acid from cysteine is encouraged due to drug resistance.

Taken together, using metabolic profiling methods coupled with robust chemometric analysis, we investigated the serum metabolic consequences of three commonly used microtubule-targeting combination chemotherapy regimens for lung cancer. Notably, the discriminatory overall metabolic readouts for the sensitive and insensitive in this study appear to be quite different from that was observed for neoadjuvant chemotherapy for breast cancer^20^, suggesting that the discriminatory metabolite factors of chemotherapy sensitivity for one type of tumor might not be transferrable for other types of tumor. The above elucidation as an effort to outline the metabolism profile of combination chemotherapy for lung cancer is of value for understanding the chemo-drug resistance, however, different discriminatory metabolites might be identified with different analysis strategy, as a recent pilot study on 25 patients before and after chemotherapy radiation has shown. Study on larger cohort is warranted for more solid conclusion.

## Materials and Methods

### Subjects and samples

The study protocol was approved by the Human Ethics Committee of Hubei Cancer Hospital, and written consent document was obtained from all healthy and patient participants at the beginning. Personally identifiable information was encrypted to protect confidentiality. All procedures involving the human subjects were carried out in accordance with the recommendations of the Helsinki Declaration.

**Figure 7:**
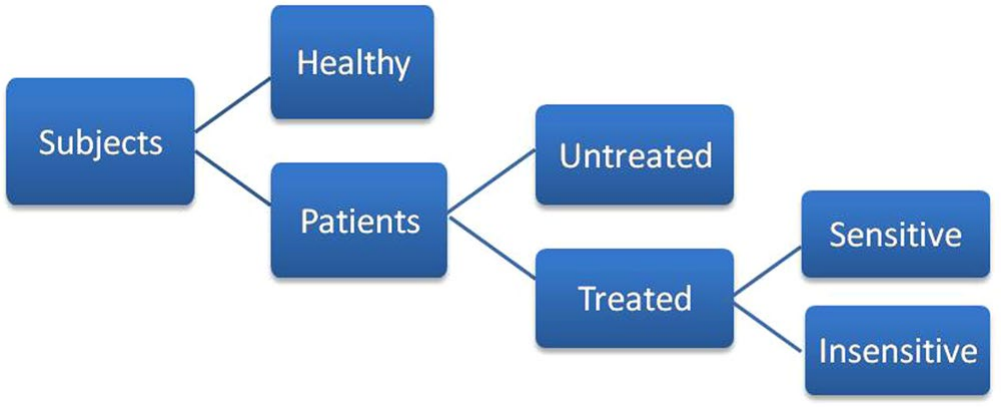
Composition of the total subjects in this study.

Of the 93 participating subjects (Fig. 7), 24 healthy people that had neither current disease nor personal history of cancer with age ranging in 32–70 years constituted our healthy control group. The other 69 lung cancer patients who had been diagnosed by imageology and pathology at Hubei Cancer Hospital from Jun. 2010 to Oct. 2012 formed our patient group (Table 1). The patient group included “untreated” subgroup and “treated” subgroup. The untreated group had 27 lung cancer patients (aged 39–74 y, average 57 y) who had been newly diagnosed and not yet gone through any treatment, while the treated group included 42 patients (aged 38–74 y, average 59 y) who had been diagnosed and undergone a kind of appropriate platinum-based combination chemotherapy as first-line treatment. The eligible patients were assessed and confirmed with histologic or cytologic clinical examination to be of SCLC or NSCLC with stage III or IV disease before the treatments.

**Table 1:** Characteristics of Controls and Patients.

	Treated Patients	New Diagnosis	Healthy Controls
Subjects	42	27	24
Average	Aged 38–74 y, Average 59 y	39–74 y, Average 57 y	32–70, Average 55 y
Gender	Male 34, Female 8	Male 20, Female 7	Male 18, Female 6
Histopathology	Adenocarcinoma 26	Adenocarcinoma 16	
	Epidermoid Carcinoma 8	Epidermoid Carcinoma 1	
	Adenosquamous Carcinoma 4	Adenosquamous Carcinoma 4	
	Bronchial Carcinoma 2	Bronchial Carcinoma 3	
	Small Cell Carcinoma 2	Small Cell Carcinoma 3	
Treatment (collected after two weeks)	GP Programme 24		
	NP Programme 14		
	DP Programme 4		
Therapeutic Effect	Sensitive 22		
	Insensitive 20		

In this study, platinum-based combination chemotherapy stands for a chemotherapy combining cisplatin with a microtubule-targeting chemo-drugs such as gemcitabine, navelbine, or docetaxelso that the observed chemotherapy-caused metabolism alteration is from DNA damage and microtubule interferences by GP programme (gemcitabine and cisplatin), NP programme (navelbine and cisplatin), or DP programme (docetaxel and cisplatin) treatment. The response to treatments was assessed carefully at the end of two cycles of any treatment. To be noted, only those patients with no previous chemotherapy treatment or with only one prior chemotherapy treatment but with a performance status 0 to 2 were included, this constrain ensures the patients had adequate organ functions.at least two cycles of. Neither gender nor age distribution had been restrained although the proportion of males is bigger than that of females among case group subjects. No gender effect on the metabolite discrimination between any groups was observed in this study.

Venous blood samples for all subjects were collected in the morning after at least 8 hours fasting. Blood (~4 ml) was acquired to a sterile universal container without anticoagulant and allowed to clot for two hours and centrifuged at 8000 r/min for 10 min at room temperature. The supernatant was harvested and stored at −80 °C until assayed. For treated patients, blood was collected at end of the second cycle of treatment.

### Sensitive and insensitive patient subjects

To assess the antitumor effect of a chemotherapy treatment, imaging-based evaluation was used to detect lesions, including chest X-ray, CT, MRI and ultrasound. Definitions of objective tumor response take into account the measurement of the longest diameter for target lesions according to WHO criteria^46^. The tumor response of treatment is then classified as: complete response (CR) - the disappearance of all target lesions; partial response (PR) -at least a 30% decrease in the sum of the longest diameter of target lesions comparing with the baseline since the treatment started; stable disease (SD)-neither sufficient shrinkage nor sufficient increase in the sum of the longest diameter since the treatment started; progressive disease (PD)-at least a 20% increase in the sum of the longest diameter of target lesions since the treatment started or the appearance of new lesions^46^. According to the pathologic assessment after treatment, therapeutic effect was divided into sensitive and insensitive to treatments. In this study, the tumor response CR, PR and SD were defined as sensitive to treatment, while the tumor response PD as insensitive to treatment. More details of subjects were listed in Table 1.

### NMR sample preparation and ^1^H NMR spectroscopy

Thawed serum samples were subjected to direct 1D &2D NMR spectra collection without dilution but with 5% D_2_O. The spectrum processing and assignment have been described previously^47,48^, and can be found in supporting information.

### Pattern Recognition Analysis

The segmented NMR datasets were imported into SIMCA-P+ (V12.0, Umea, Sweden) for multivariate data analysis. PCA was first performed to examine group clustering and possible outliers using mean-center scaling. OPLS-DA was further performed with the unit-variance scaled NMR data as the X-matrix and group information as the Y-matrix^49^, and the built models were assessed with the 7-fold cross-validation method (R^2^X as explained variations and Q^2^ as model predictability). The model was further validated with CV-ANOVA approach (with p < 0.05 as significant)^50^. The back-transformed loadings were plotted with color-scaled correlation coefficients of variables using an in-house script. The color-coded correlation coefficients showed the intergroup differentiation metabolites, with the “warm” color variables reflecting significant difference contribution.

### Receiver Operating Characteristic (ROC) Curve

To further evaluate the predicative ability of OPLS-DA models, receiver operating characteristic (ROC) curve was acquired from Y-predicted values. Area under the ROC curve (AUC) was calculated using the performance curve algorithm from SPSS 18.0 (SPSS Inc., Chicago, IL, USA).

## Electronic supplementary material

supporting materials
