# Supplementary material for: Serum Metabolic Profile Alteration Reveals Response to Platinum-Based Combination Chemotherapy for Lung Cancer: Sensitive Patients Distinguished from Insensitive ones
Source: Sci Rep. 2017 Dec 13;7:17524. doi: 10.1038/s41598-017-16085-y (PMC5727535; doi:10.1038/s41598-017-16085-y)
Supplement: Supplementary file 1 — supporting materials [file 41598_2017_16085_MOESM1_ESM.pdf]

Supporting Information

Serum Metabolic Profile Alteration Reveals Response to Platinum-Based Combination Chemotherapy for Lung Cancer： Sensitive Patients Distinguished from Insensitive ones

Shan Xu[1,4,5,#], Yanping Zhou[1,#], Hui Geng[3,#], Dandan Song[1], Jing Tang[2], Xianmin Zhu[2], Di Yu[5,6], Sheng Hu[2,*], and Yanfang Cui[1,6,*]

1. *Key Laboratory of Pesticide and Chemical Biology, Ministry of Education, Central China Normal University, Wuhan 430079, P. R. China*
2. *Department of Medical Oncology, Hubei Province Tumor Hospital, Wuhan 430079, P.R. China*
3. *Department of Life Sciences, Central China Normal University, Wuhan 430079, P. R. China*
4. *Tongji Medical College, Huazhong University of Science and Technology, Wuhan 430030, P.R. China*
5. *CAS Key Laboratory of Magnetic Resonance in Biological Systems, State Key Laboratory of Magnetic Resonance and Atomic and Molecular Physics, National Centre for Magnetic Resonance in Wuhan, Wuhan Institute of Physics and Mathematics, University of Chinese Academy of Sciences, Wuhan 430071, China*
6. *Department of Biochemistry and Molecular Biology, Biomedicine Discovery Institute, Monash University*

# The authors contribute equally

*Corresponding author: Dr. Yanfang Cui Email: yfcui@mail.ccnu.edu.cn, Tel: +86-27-6786 7953

Dr. Sheng Hu Email: ehusmn@163.com Tel: +86-27-87670351

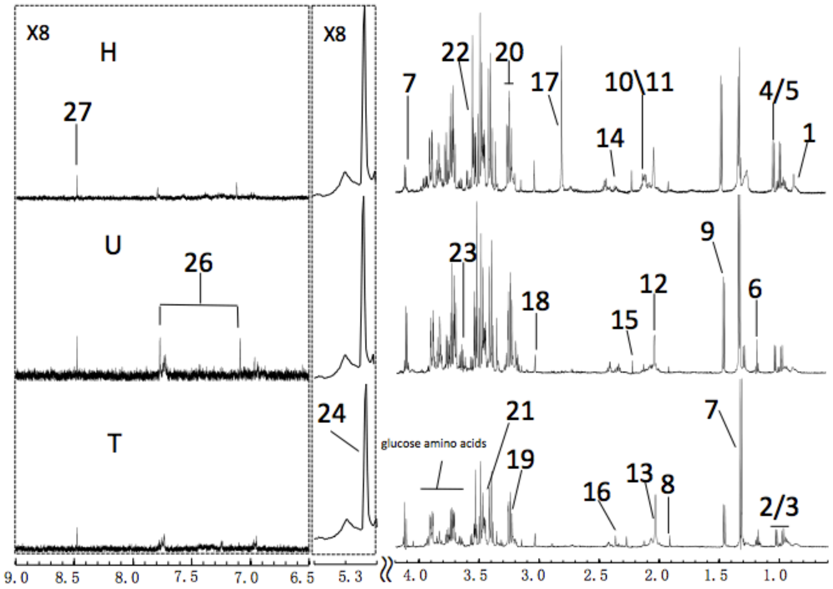

Figure S1. 1D NMR spectrum 600 MHz ^{1}H NMR spectra for human serum of Healthy (C), Untreated (U) and Treated (T) groups. 1. Lipid, 2.Isoleucine, 3. Leucine, 4.Valine, 5.Isobutyrate, 6.3-hydroxybutyrate, 7.Lactate, 8.Acetate, 9.Alanine, 10.Glutamate, 11.Methionine, 12.Glycoprote, 13.Proline, 14.Glutamine, 15.Acetoacetate , 16. Pyruvate, 17.Trimethylamine, 18.Creatinine, 19.Choline , 20. Taurine, 21.TMAO, 22. Glycine 23. Myo-inositol, 24.α-glucose, 25.β-glucose, 26.Histidine, 27.Formate.

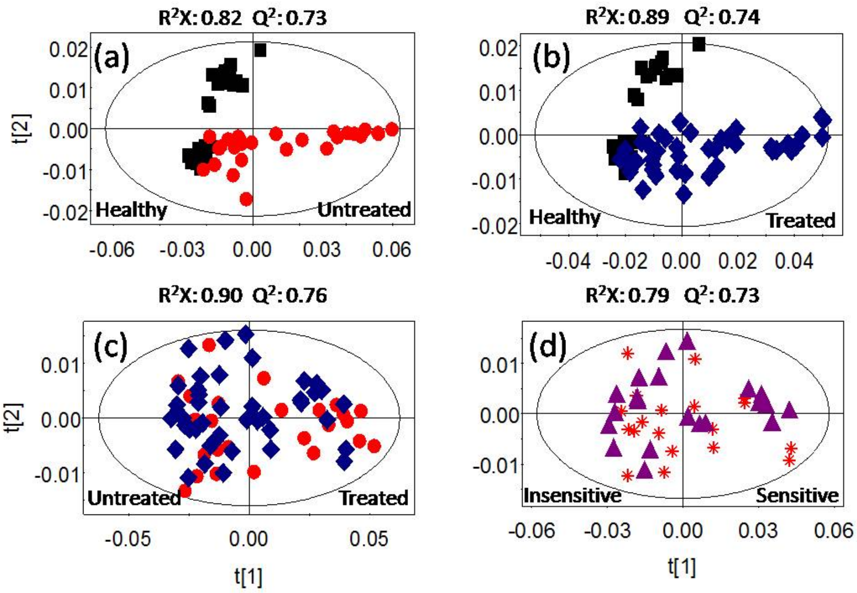

Figure S2. PCA scatter diagram between groups of healthy control (■), Untreated (●), Treated (◆), Insensitive(▲) and Sensitive (*).

Table S1. NMR assignment for the metabolites found in human serum.

	metabolite	ppm		chemical bond
1	Lipid	0.88	br	CH3CH2CH2C
		1.26	br	CH3CH2CH2
2	Isoleucine	0.94	d	δ-CH3
		1	d	β- CH3
3	Leucine	0.96	t	δ- CH3
		1.71	m	β- CH2, γ- CH
4	Valine	0.98	d	CH3
		1.03	d	CH3
		3.57	d	α- CH
5	Isobutyrate	1.07	d	CH3
6	3-hydroxybutyrate	1.2	d	γ- CH3
		2.3	m	Half α- CH2
7	Lactate	1.33	d	CH3
		4.11	q	CH
8	Acetate	1.92	s	CH3
		1.92	s	CH3
9	Alanine	1.46	d	CH3
10	Glutamate	2.03	m	Half β- CH2
11	Methionine	2.13	s	S-CH3
12	Glycoprotein(acetyls)	2.04	s	NHCOCH3
13	Proline	2.06	m	Half β- CH2
		3.34	m	Half δ-CH2
14	Glutamine	2.08	m	Half β- CH2
		2.35	m	Half γ- CH2
		2.41	m	Half γ- CH2
15	Acetoacetate	2.22	s	CH3
16	Pyruvate	2.37	s	CH2
	Dimethylamine	2.72	s	CH3
17	Trimethylamine	2.81	s	CH3
18	Creatinine	3.03	s	CH3
	Creatine	3.93	s	CH2
19	Choline	3.2	s	N(CH3)3
20	Taurine	3.25	t	CH2NH
		3.41	t	CH2SO3
21	TMAO	3.26	s	CH3
22	Glycine	3.55	s	CH2
23	Myo-inositol	3.65	dd	H4,H6
		3.54	dd	H4,H6
24	α-glucose	3.42	t	H4
		3.53	dd	H2
		3.71	t	H3
		5.23	d	H1
25	β-glucose	3.4	t	H4

		3.46	m	H5
		3.47	t	H3
		4.64	d	H1
		3.24	dd	H2
26	Histidine	7.03	s	H4
		7.73	s	H4
27	Formate	8.45	s	CH

Supporting information for materials and methods

Sample preparation and High-resolution ^{1}H NMR spectroscopy

Serum samples were thawed at room temperature, and then 400 μl aliquots of serum were reconstituted into 500 μl with D2O for deuterium lock purpose. The mixed serum solution was transferred into 5 mm NMR tubes. For all samples, the standard one-dimensional (1D) 1H pulse sequence and Carr–Purcell–Meiboom–Gill (CPMG) pulse sequence both with water presaturation were respectively applied at 599.660 MHz with the 90° pulse length (about 10 μs). A total of 64 free induction decays (FIDs) were collected into 16K data points with a relaxation delay of 4 s.

To facilitate resonance assignment, a series of 2D NMR spectra including 1H−1H correlation spectroscopy (COSY), 1H−1H total correlation spectroscopy (TOCSY), 1H J-resolved spectroscopy (JRES), 1H−13C heteronuclear multiple bond correlation spectroscopy (HMBC) and 1H−13Cheteronuclear single quantum correlation spectroscopy (HSQC) were acquired and processed as previously described[1,2.]

Data processing of the NMR spectra

All 1D data sets were zero-filled to 32,000 data points, and exponential line broadening of 0.2 Hz was applied before Fourier transformation. Chemical shifts were referenced to H-1 of α-glucose (δ 5.23) for 1H.All NMR spectra were phased and manual-baseline corrected using Topspin software package (V3.0, BrukerBiospin, Germany) prior to data reduction. Subsequently the spectral ranges of δ 0.50–8.50 were segmented into bins between with the bin size of 0.004 ppm using AMIX software package (V3.9.5, BrukerBiospin, Germany). The residual water signal in regions of δ4.20-5.20 was discarded for eliminating the effects of imperfect water saturation. The data of each segment were normalized to the sum of total integrals of each spectrum, in order to compensate for the differences in overall concentration between individual samples. To calculate the relative quantification (in form of [Cm–C0]/C0, where Cm stood for the peak areas of a particular metabolite signal in treated or untreated group, C0 for that in healthy group) for some specific metabolites, the least overlapped characteristic signal of a metabolite was integrated and the integrals standing for the metabolite concentrations were used in the calculation.

References

1 Dai, H., Xiao, C., Liu, H. & Tang, H. Combined NMR and LC-MS analysis reveals the metabonomic changes in Salvia miltiorrhiza Bunge induced by water depletion. *J. Proteome Res.* **9**, 1460-1475 (2010).

2 Dai, H., Xiao, C., Liu, H., Hao, F. & Tang, H. Combined NMR and LC-DAD-MS analysis reveals comprehensive metabonomic variations for three phenotypic cultivars of Salvia Miltiorrhiza Bunge. *J. Proteome Res.* **9**, 1565-1578 (2010).
